# A problem‐based approach to understanding public support for referendums

**DOI:** 10.1111/1475-6765.12368

**Published:** 2020-01-07

**Authors:** HANNAH WERNER, SOFIE MARIEN, ANDREA FELICETTI

**Affiliations:** ^1^ Centre for Political Research KU Leuven Belgium

**Keywords:** democracy, referendums, European Social Survey (ESS), preferences

## Abstract

A prominent explanation of widespread popular support for referendums is dissatisfaction with the functioning of representative democracy. In this article, the aim is to gain a better understanding of how dissatisfaction affects support for referendums. Drawing on previous research, it is argued here that citizens follow a problem‐based approach in their support for referendums, in that referendums are considered a suitable solution to address some specific problems in a political system but not all. Survey data from the 2012 European Social Survey (29 countries; N = 37,070) is used to show that citizens’ expectations towards and evaluations of representatives relate to support for referendums. In particular, dissatisfaction with the ability of governments to listen to their citizens is associated with higher support for referendums. In contrast, citizens dissatisfied with the government's ability to lead are less supportive of referendums. Furthermore, the relationship between dissatisfaction with governments’ ability to listen varies across countries depending on the level of experience with decision making via referendum. In countries where referendums are used more often, the expectation of referendums being able to solve the problem of unresponsive government is weaker. This study offers important insights into the different ways in which preferences and evaluations of representative practices relate to popular support for referendums.

## Introduction

Public opinion surveys reveal strong support for direct decision making by citizens (ISSP 2016; ESS 2012). While there is no consensus on how to interpret or explain this popular support, dissatisfaction seems to lie at the heart of extant explanations. Literature focusing on the postmaterial value shift in society argues that citizens are dissatisfied with the limited ability to provide input to democratic government under current institutional arrangements. Hence, support for referendums is interpreted as a desire for more opportunities for a voice in political decision‐making processes (Inglehart 2008; Dalton & Welzel 2014; Norris 1999, 2011; Bowler et al. 2007). However, this interpretation of a widespread genuine desire for participatory processes has been challenged, most prominently by Hibbing and Theiss‐Morse (2002). They interpret the support for direct decision‐making practices as a reflection of dissatisfaction with representatives. Because representatives cannot be trusted, citizens feel they need to intervene, although that is not their preferred course of action (Hibbing & Theiss‐Morse 2002; Dalton et al. 2001; Bowler et al. 2007; Schuck & De Vreese 2015). Empirically, dissatisfaction with representative democracy emerges as a core driver of support for referendums, as documented by recent survey research (Anderson & Goodyear‐Grant 2010; Dalton et al. 2001; Webb 2013; Collingwood 2012; Bengtsson & Mattila 2009; Schuck & De Vreese 2015; for an exception, see Bowler et al. 2007). Yet the meaning of this link remains unclear. What kind of dissatisfaction causes citizens to desire this alternative decision‐making arrangement?

In this study, we aim to contribute to a better understanding of popular support for referendums across Europe. Inspired by Mark Warren's (2017) problem‐based approach to democracy, we argue that citizens employ a pragmatic approach to referendum support. There can be different problems with representative democracy and citizens see referendums as a potential solution to some of them, though not all. We illustrate how this approach plays out by focusing on how preferences for and evaluations of representative practices relate to support for direct decision making via referendums.

To this end, we connect to the literature on representation and draw on the well‐known distinction between delegate and trustee representation. Following the delegate view representatives should listen and follow public opinion closely. In the trustee view, representatives have greater freedom to follow their own judgement and lead the way. We expect that preferences for these different models of representation are related to opinions about the role of citizens in decision‐making processes. If listening is strongly valued, then support for more opportunities for voice in political decision‐making processes and interventions by citizens will be higher. Furthermore, we argue that dissatisfaction with representation relates to support for referendums in different ways – that is, dissatisfaction with the government to fulfil its function to listen to citizens is expected to relate to high levels of support for referendums. On the contrary, we do not expect such a relationship to occur with regard to dissatisfaction with the government's ability to lead because referendums are not a solution for this specific problem. Finally, we investigate whether the relationship between dissatisfaction with the listening function of government and support for referendums varies across countries depending on the level of experience a country has with referendums as a decision‐making tool. It could well be that referendums are considered more or less suitable to tackle the problem of unresponsive government depending on whether and to what extent a society has experienced referendums in practice.

We test these expectations using regression analyses on the 2012 European Social Survey (ESS) data. The sixth round of this biannual survey is particularly suited for this test as it includes questions about respondents’ conceptions of the government's role (listening or leading) as well as its performance on this criterion. Interviews were conducted in 29 countries covering information on 37,235 respondents in total. The data is cross‐sectional, which limits the possibility for causal inference, but it does allow for a first insight into the relations between preferences and evaluations of government responsiveness and support for referendums in a large number of European countries.

The results show that citizens who prefer the government to listen rather than lead are more supportive of referendums. Importantly, dissatisfaction with the listening function of the government relates positively to support for referendums, whereas dissatisfaction with the government's ability to lead is negatively related. This supports our argument that only specific types of dissatisfaction are associated with higher support for referendums. Furthermore, we find that the relationship between dissatisfaction with listening and support for referendums is weaker in countries with more experience with referendums. These findings show that direct decision‐making practices could address dissatisfaction with some representative practices, but not all kinds of dissatisfaction can be addressed by more direct interventions featuring citizens. We need a more nuanced understanding of how dissatisfaction affects support for referendums. By connecting normative expectations and evaluations of representation to support for referendums this study provides an important step in this direction.

## Dissatisfaction and support for referendums

There is a vibrant literature that aims to explain popular support for referendums (Bowler et al. 2007; Donovan & Karp 2006), other forms of citizen involvement (Neblo et al. 2010; Webb 2013), and different models of citizenship and democracy (Allen & Birch 2015; Bengtsson & Mattila 2009; Coffé & Michels 2014; Font et al. 2015). In this literature, normative ideals on how democracy should function and dissatisfaction with current democratic performance have attracted substantial attention as potential drivers of support for referendums.

In the two most prominent approaches to process preferences, dissatisfaction takes a central yet different role. First, the cognitive mobilisation thesis describes the emergence of critical, assertive or self‐actualised citizens who are dissatisfied with the limited role that current representative structures entail for citizens to take matters in their own hands. This sentiment is argued to emerge from a shift towards postmaterialist and emancipative values and rising levels of education and information access (Inglehart 2008; Dalton & Welzel 2014; Norris 1999, 2011). Second, for the stealth democracy thesis, the argument goes that citizens are not genuinely supportive of more participation opportunities but rather want government to quietly fulfill its tasks without much involvement or even monitoring by citizens. Only when citizens are dissatisfied with how politicians do their job will they demand means to take over control, such as through referendums (Hibbing & Theiss‐Morse 2002; Dalton et al. 2001; for an overview of both approaches, see Bowler et al. 2007).

While both strands of the literature put dissatisfaction central, its nature and specific role in driving support for participatory processes is rarely specified. Furthermore, empirical work has mostly relied on general indicators such as satisfaction with democracy or political trust. Interestingly, the consistent effect of dissatisfaction on support for referendums (e.g., Schuck & De Vreese 2015; Webb 2013; Bengtsson & Mattila 2009) has mostly been interpreted as support for the stealth democracy thesis. Hence, the dissatisfaction effect has been considered evidence that citizens do not genuinely desire participation, while high levels of support are seen as expressions of their overall frustration with politicians.

In this article, we aim to gain a better understanding of the relationship between (different types of) dissatisfaction and support for referendums. In addition to the more abstract and value‐oriented considerations about the ability of referendums to counter diffuse dissatisfaction with political institutions, we argue that support for referendums is also affected by pragmatic considerations. In a recent seminal contribution, Warren (2017) has argued for a problem‐based approach to democracy, in which we should refrain from thinking about democracy only in terms of some democratic models we favour. Rather, we should consider also which institutions are capable of tackling different democratic problems in political systems. Inspired by Warren's call, we seek to show that pragmatic considerations actually play a role also when it comes to citizens’ support for referendums. In effect, referendums could offer pragmatic solutions for specific problems that citizens see in contemporary politics. This approach allows us to see that dissatisfaction does not always lead to support for referendums. Referendums might be deemed suitable for dealing with some types of dissatisfaction but not others.

It is important to note that our approach does not contradict existing approaches to understanding process preferences, such as the cognitive mobilisation thesis or the stealth democracy thesis. Rather, we assume that individuals’ preferences for different procedural arrangements have different components: some of them value‐based and stable, and others context‐dependent and dynamic (see also Werner 2019).

We illustrate our argument by connecting our research on process preferences to the literature on representation. The classic distinction between delegate and trustee as formulated by Burke is useful in this regard (see, e.g., Fox & Shotts 2009). In the delegate model, representatives need to follow citizens’ instructions and closely monitor and represent the opinions of the electorate. In contrast, trustee representation refers to politicians acting autonomously and on behalf of the public interest. For this study, we adopt Bowler's ideas of ‘government as listeners’ and ‘government as leaders’ that he devised to mark the distinction between the approaches pursued by members of parliament, which the delegate and trustee labels have traditionally been used for. As Bowler (2017) recently showed, citizens hold diverging views as to whether politicians should act as listeners or as leaders (see also Barker & Carman 2012). Furthermore, citizens can be dissatisfied with the government's ability to listen to its constituents as well its ability to lead. Accordingly, we pose the following research question:

*RQ1*: How do citizens’ preferences and evaluations of representative practices affect popular support for referendums?

We argue that both preferences for different representation ideals as well as their evaluation relate differently to support for referendums. First, we expect citizens who prefer the government to listen to be more in favour of decision making via referendums than citizens who prefer the government to lead. Both a responsive government and implementing a referendum result approach the ideal of policy following public opinion. Hence, if citizens prefer one, they are likely to prefer the other. This is different for citizens who think the government should lead. To these citizens, responsibility is more important than responsiveness; representative government is not expected to rule by following the sway of public opinion. During elections governments would be dismissed if they perform poorly, but in the meantime, they should be free to follow their own initiative. Hence, support for the use of direct decision‐making practices is expected to be lower for this group of citizens. This expectation is expressed in the following hypothesis:

*H1*: People who believe the government should closely listen and respond to public opinion are more supportive of referendums than citizens who prefer the government to lead.

Second, we expect dissatisfaction with these different functions to affect support for referendums differently. If citizens are dissatisfied with the government's ability to listen to the public, support for referendums is likely to be high. This is because referendums are likely considered a direct solution to a deficit in perceived responsiveness. If the government does not voluntarily take public opinion into account, then referendums are a suitable strategy to make their voices heard and be translated into policy. In contrast, when citizens are dissatisfied with the government's performance on leading, support for referendums is not expected to increase. If it is the government's job to take decisions and to lead, then direct interventions by citizens (e.g., via referendums) do not represent a solution to this particular problem. In sum, whereas we expect dissatisfaction with the listening function of a government to be related to higher support for referendums, we expect no such relationship for dissatisfaction with the leading function of a government. Accordingly, we formulate the following hypotheses:

*H2a*: People who are more dissatisfied with the government's ability to listen to public opinion are more supportive of referendums than people who are less dissatisfied.
*H2b*: People who are more dissatisfied with the government's ability to lead are not more supportive of referendums than people who are less dissatisfied.

These expectations have not been extensively tested within the rich literature on process preferences. Citizens’ expectations of representation and the respective evaluations have rarely been included in efforts to explain support for direct democratic practices. Yet, there are indications that support for referendums relates to preferences and evaluations of responsiveness. Bowler et al. (2003) focus on legislators’ responsiveness using data from Californian voters. They show that a delegate view is associated with more support for referendums than a trustee view. Allen and Birch (2015) show that citizens who are more critical of politicians’ integrity and responsiveness tend to express support for greater levels of popular involvement in political decision making. In a recent article, Fernández‐Martinez and Font Fábregas (2018) connected people's views on responsiveness to their support for referendums. Their analyses show that for some people these views are strongly connected, while this is not the case for others. We contribute to this literature by linking citizens’ expectations of representation and their evaluations thereof to support for referendums.

In addition, we explore how the relationship between dissatisfaction with the listening function and support for referendums varies across country context. We are especially interested in the role that experience with referendums plays in this regard. In some countries, supporting referendums is a rather hypothetical exercise because the country has little practical experience with national‐level referendums, either because they are not legally possible or because they occur only rarely (for an overview, see Setälä 2006; Bjørklund 2009). In other countries, however, referendums are used more frequently and hence support for referendums refers to an existing decision‐making tool. It may be possible that citizens in contexts where experience with referendums is low have different expectations of what kind of problems referendums can solve compared to contexts where such experience is high. Possibly, referendums are an instrument that looks only appealing as a solution for unresponsive governments in the abstract and is considered less of a solution for this problem once it is in place. On the other hand, the experience with referendums might also lead to higher confidence in them to counteract a lack of government responsiveness. Since we have no grounded expectation of the direction of this relationship, we posit the following research question without formulating specific hypotheses:

*RQ2*: Does country‐level experience with referendums moderate the relationship between dissatisfaction with governments’ ability to listen and support for referendums?

## Data

We use data from the European Social Survey, Round 6 (ESS 2012). The interviews were conducted face‐to‐face in 2012 and 2013. They are highly suitable for our research because the survey included a new module called ‘Understandings and evaluations of democracy’. This module asked several questions on how citizens think democracy should work and also on how they evaluate the performance of their respective political system (Ferrín & Kriesi 2016). The dataset includes 54,673 interviews in 29 countries. After excluding missing data, our analytic sample consists of 37,070 European citizens. For country‐level information on experience with referendums, we use the C2D (Centre for Research of Direct Democracy 2019) and the SUDD (2019) databases. The data are analysed using multilevel regressions. Following ESS guidelines, we apply post‐stratification weights.

Our outcome variable is support for referendums. We use one of the items of the battery on understandings of democracy that reads: ‘How important it is for democracy that citizens have the final say on political issues by voting directly in referendums?’ The answers are measured on a 0 to 10 (11‐point) scale.

### Explanatory variables

Round 6 of the ESS includes a question that taps into respondents’ preferences regarding government responsiveness. The question asks respondents to choose between two options regarding what the interviewee ‘thinks is best for democracy in general’: (a) The government should change its planned policies in response to what most people think; and (b) the government should stick to its planned policies regardless of what most people think. There is a coding for ‘it depends on circumstances’ and ‘don't know’ answers that are not mentioned by the interviewer.

Subsequently, respondents are asked to what extent government fulfills this function today (11‐point scale from 0 ‘never’ to 10 ‘always’). As a result, we can gain insight into the extent to which these different views on government responsiveness are shared in European societies and how well governments are performing according to these expectations. The question on adherence to the listener or leader view served as a filter question. Respondents who indicated they adhere to the ‘listener’ model were only asked about evaluation of this function. Respondents who indicated they adhere to the ‘leader’ model were only asked about evaluation of this function. Respondents who indicated their opinions ‘depend’ or they ‘don't know’ were questioned on the listener function.^1^ We recoded the evaluation question so that high values indicate dissatisfaction with government performance.

### Moderator

To grasp country‐level experience with referendums, we construct a variable based on the C2D database (C2D 2019). This database, hosted by the ETH Zurich, contains information on national‐level referendums for several countries across the globe from 1970 until today (it is updated regularly). The data we use stem from May 2019. The database contains information for all countries in our dataset with the exception of Kosovo. For this country, we relied on data from the SUDD database (SUDD 2019). Both databases are regularly used in scholarly research on referendums (e.g. Mendez & Germann 2018).

### Control variables

We include measures that are known to affect support for referendums: education (Coffé & Michels 2014), age, sex, left‐right placement, political interest and generalised trust. Further, we also include satisfaction with democracy to see whether the specific types of dissatisfaction we focus on in this study play out differently than the commonly used general indicator for dissatisfaction. As we are also measuring evaluations of the government in each country, we include a dummy variable on whether or not the interviewee voted for an incumbent party (i.e., the party is included in the cabinet).

## Results

As expected, support for referendums in Europe is high. The mean score is 8.27 on a 0–10 scale, with a standard deviation of 2.04. While this is a high mean, there are individual variations that are interesting to explore further. There is also quite some diversity in respondents’ views on how their government should act. In the sample, the majority of respondents expects the government to closely listen and respond to public opinion (66 per cent). Another 17 per cent of the sample prefers the government to act as a leader rather than to shift policies in line with public opinion. Another 12 per cent says it depends, while 5 per cent say they do not know. While the listener view is clearly widely shared, it is not the only preferred model among European citizens. When recoding the data to the two main models ‘listener’ and ‘leader’, the distribution is approximately 80 per cent holding the listener view and 20 per cent holding the leader view (in the full sample and in the analytical sample).^2^

**Figure 1:**
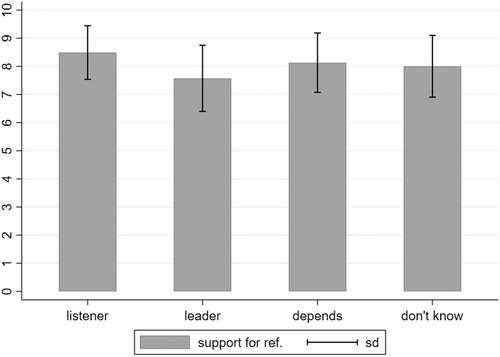
Support for referendums across representative ideals. Notes: N = 54,515. Source: ESS (2012).

Given the nested nature of the data in 29 countries and our interest in cross‐level interactions we employ multilevel regression modelling using Stata 14 (www.stata.com) to testing all hypotheses. Turning first to *H1*, we predicted that citizens who prefer the government to listen exert higher support for referendums than citizens who prefer the government to lead. When simply plotting the means (see Figure 1), this seems to be indeed the case.

The respondents preferring the government to first and foremost listen have higher average levels of support for referendums than the respondents who prefer government to lead (*M_Listen_* = 8.49, *SD* = 1.90; *M_Lead_* = 7.57, *SD* = 2.35). The multilevel regression model confirms that listeners have a significantly higher level of support than leaders. The full model can be found in the Online Appendix, Table A3.

Turning to our main hypotheses, *H2a* and *H2b*, we investigate to what extent dissatisfaction with the listening and leading functions of the government is associated with support for referendums. As model I in Table 1 shows, dissatisfaction with the listening function of the government relates positively and significantly to support for referendums, even when controlling for general dissatisfaction.^3^ This means people who are dissatisfied with the government's ability to listen to citizens’ views are also in favour of decision making via referendums. Turning to dissatisfaction with the leading function, we expected no such relationship. However, the data indicates a significant negative relationship, as can be seen in model II. Accordingly, citizens who are dissatisfied with the government's ability to lead are even less supportive of referendums than those who are satisfied with its performance on this particular function. Apparently, referendums decline in popularity when other problems, such as leading, become more pressing in the eyes of citizens.

**Table 1:** Multilevel regression explaining support for referendums

	Model I			Model II		
	Coef.	p	Robust SE	Coef.	p	Robust SE
Dissatisfaction listening function	**0.07**	**0.000**	**0.01**			
Dissatisfaction leading function				**–0.07**	**0.003**	**0.02**
Dissatisfaction (combined)						
Representation ideal						
Dissatisfaction × Representation model						
Satisfaction with democracy	–0.04	0.010	0.02	–0.02	0.381	0.03
Voted for incumbent	0.01	0.740	0.04	–0.08	0.390	0.09
Generalised trust	0.01	0.539	0.01	–0.04	0.005	0.01
Political interest	0.16	0.000	0.02	0.08	0.350	0.09
Left‐right placement	0.01	0.636	0.02	0.03	0.299	0.03
Gender	0.01	0.860	0.03	–0.20	0.001	0.06
Education						
Lower secondary	0.42	0.000	0.07	–0.05	0.745	0.16
Upper secondary	0.51	0.000	0.10	0.18	0.013	0.07
Advanced vocational	0.44	0.000	0.09	–0.20	0.362	0.22
University degree	0.34	0.000	0.07	–0.57	0.000	0.10
Age	0.01	0.000	0.00	0.00	0.144	0.00
Constant	7.24	0.000	0.21	8.16	0.000	0.27
Variance (intercept)	0.13	0.000	0.03	0.32	0.000	0.09
Wald Chi^2^	3687.96	0.000		482.22	0.000	
N	28,849			8,221		

Notes: Non‐standardised coefficients are presented, coefficients relating to hypotheses are printed in bold. Weights are applied.

Source: ESS (2012).

**Figure 2:**
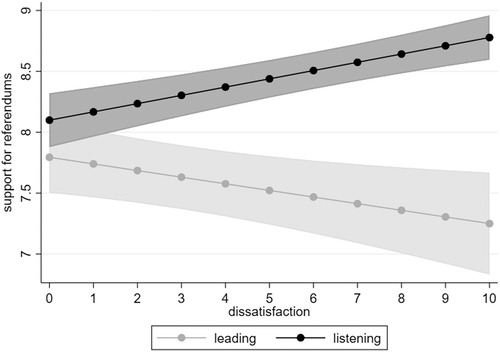
The effects of different types of dissatisfaction on support for referendums. Notes: Estimates are the result of a multilevel regression (see Table 1, model III). N = 37,235. Source: ESS (2012).

These findings confirm *H2a* and partially confirm *H2b*. As theorised, not all types of dissatisfaction are related to increased support for referendums. As the data show, some types of dissatisfaction can even have the reverse effect. We visualise the different effects of these two different types of dissatisfaction in Figure 2.

**Figure 3:**
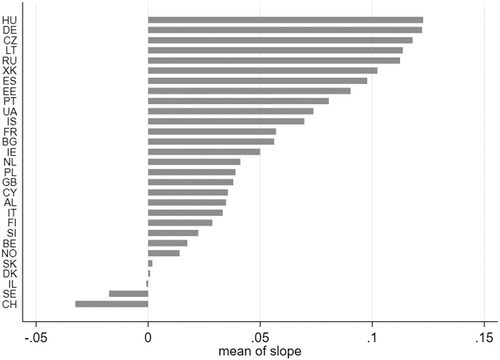
Slope coefficients for the effect of dissatisfaction with listening on support for referendums across countries. Notes: Estimates are the results of a multilevel regression with random slopes (Table 2, model I). N = 28,849. Weights could not be applied for this visualisation. Source: ESS (2012).

Turning to the second research question, we aim to investigate the relationship between dissatisfaction with the listening function and support for referendums, taking contextual factors into account. In a first step, we run a random slope model to see if there is variation of the strength of this association across countries. As model I in Table 2 shows, the significant variance and the reduced Wald Chi^2^ indicate that this is the case, although the reduction in Wald Chi^2^ seems moderate. Figure 3 visualises the different slope coefficients for the different countries under study.

**Table 2:** Random slopes models explaining cross‐country variation

	Model I			Model II			Model III		
	Coef.	p	Robust SE	Coef.	p	Robust SE	Coef.	p	Robust SE
*Country level*									
experience with referendums				0.01	0.001	0.00	0.01	0.000	0.00
*Individual level*									
Dissatisfaction listening function	**0.05**	**0.000**	**0.01**	0.05	0.000	0.01	0.07	0.000	0.01
Dissatisfaction listening × Experience with referendums							**0.00**	**0.001**	**0.00**
Satisfaction with democracy	–0.04	0.013	0.01	–0.04	0.011	0.02	–0.04	0.009	0.02
Voted for incumbent	0.01	0.750	0.04	–0.02	0.649	0.03	–0.02	0.543	0.03
Generalised trust	0.01	0.504	0.01	0.01	0.486	0.01	0.01	0.465	0.01
Political interest	0.16	0.000	0.02	0.15	0.000	0.02	0.15	0.000	0.02
Left‐right placement	0.01	0.575	0.02	0.01	0.842	0.03	0.01	0.839	0.03
Gender	0.00	0.893	0.03	0.01	0.871	0.04	0.01	0.838	0.04
Education									
Lower secondary	0.41	0.000	0.07	0.43	0.000	0.08	0.43	0.000	0.09
Upper secondary	0.52	0.000	0.10	0.52	0.000	0.11	0.52	0.000	0.11
Advanced vocational	0.43	0.000	0.09	0.39	0.000	0.10	0.39	0.000	0.10
University degree	0.34	0.000	0.08	0.32	0.000	0.09	0.32	0.000	0.09
Age	0.01	0.000	0.00	0.01	0.000	0.00	0.01	0.000	0.00
Constant	7.33	0.000	0.20	7.23	0.000	0.23	7.19	0.000	0.24
Variance (intercept)	0.16	0.000	0.04	0.14	0.000	0.038	0.13	0.000	0.04
Variance (slope)	0.00	0.000	0.00	0.00	0.000	0.000	0.00	0.000	0.00
Wald Chi^2^	3122.45	0.000		2444.53	0.000		2548.69	0.000	
N	28,849			25,881			25,881		

Notes: Estimates are the result of a multilevel regression. Non‐standardised coefficients are presented. Weights are applied. In models II and III Switzerland is excluded from analysis.

Source: ESS (2012).

In a second step, we try to explain this variance by including national‐level experience with referendums as a moderator. Switzerland presents a complication in this regard. Given the enormous amount (i.e., 645) of referendums that have been held there on the national level since 1970, this case is likely to distort the analysis (average across all other countries: 13). We address this problem by running four types of models: (1) excluding Switzerland (presented here), (2) including Switzerland with its true score, (3) including Switzerland capped at a score of 100, and (4) including Switzerland and applying a log transformation of the experience variable. The results of models 2, 3 and 4 largely confirm the results and are presented in Table A4 in the Online Appendix.

**Figure 4:**
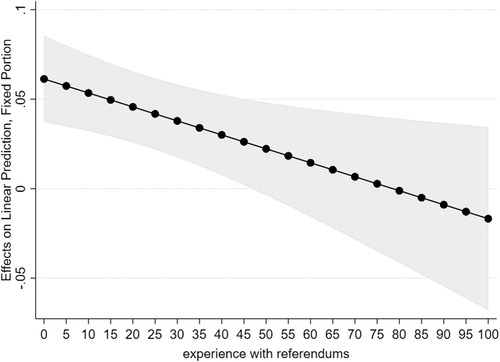
The marginal effect of dissatisfaction with listening function on support for referendums across experience with referendums. Notes: Estimates are the result of a multilevel regression (see Table 2, model III). Experience with referendums presents that amount of referendums that have been held on the national level since 1970. Switzerland is excluded from analysis.

As can be seen in model II, the interaction coefficient turns out significant and negative. This means that in countries with higher levels of experience with referendums, the relationship between dissatisfaction with the listening function and support for referendums is weaker than in countries with lower levels of experience. Figure 4 visualises this interaction in a marginsplot. As can be seen, the model predicts dissatisfaction with listening to cease to be positively associated with support for referendums after about 47 referendums since 1970, approximately ten referendums per year. Most countries in the sample score far below that threshold.

Interestingly, experience with referendums as such has a very small positive effect or no significant effect on support for referendums, depending on the specifications of the Swiss case (Online Appendix, Table A5). Hence, it is not the case that experience with referendums lowers support, but it is specifically the explanatory power of dissatisfaction with listening that depends on country‐level experience. This indicates that referendums are a tool that looks appealing as a solution for unresponsive governments in the abstract, but the available experiences with referendums to date seem to make people more critical about the potential of referendums to address specific responsiveness problems.

## Robustness checks

We conduct a range of additional checks to assess the robustness of these findings. First, we want to address the specific feature of the survey design that the sample was split based on whether respondents preferred the listener or the leader model. In our main model we find that dissatisfaction with the listening function relates positively with support for referendums whereas dissatisfaction with the leading function relates negatively. However, both dissatisfaction questions were asked to different subgroups of people. Potentially, there are two explanations for this effect. First, in line with our argument, different types of dissatisfaction relate differently to support for referendums. Second, it is also possible that for citizens that have different representation ideals, dissatisfaction in general relates differently to support for referendums.

To address which of the two explanations is valid, we test whether the general relationship between dissatisfaction and support for referendums is different for citizens who supported the leader or the listener model. If we find a significant interaction, this indicates that dissatisfaction matters differently for support for referendums depending on what representation style respondents preferred. If we do not find such an interaction, this indicates that it is the specific type of dissatisfaction (government fails to listen or to lead) that is associated with support for referendums. To this end, we run a moderation analysis where we interact citizen preferences on representation with satisfaction with democracy. We find no interaction effect, nor do we find one for other indicators of dissatisfaction, such as satisfaction with government and political trust (see Online Appendix, Table A6). Accordingly, it seems that the link between dissatisfaction and support for referendums depends on the specific political function that citizens are dissatisfied about. If the government does not listen, referendums are considered a suitable solution for this particular problem. If the government does not lead, referendums do not tackle the problem in the eyes of citizens.

Second, we test whether dissatisfaction with other aspects of the political system are positively related to support for referendums. To this end, we run the analysis with evaluations of the impartiality of the courts, of the government's success in fighting poverty, and of the media's ability to provide factual information to judge the government. For all three we would expect no association with support for referendums because referendums are hardly a solution to any of these problems. Indeed, for the evaluations of court impartiality and government success in tackling poverty, we find no significant effect. However, for the issue of unbiased media coverage we find a negative significant effect, indicating that referendums are even less desirable when one is dissatisfied with the functioning of the media (Online Appendix, Table A7). This finding again confirms that it is the type of dissatisfaction rather than the subsamples that drive our main effects.

Third, we also conduct our main analyses without general satisfaction with democracy as a control to ensure that it is not the relationship between the different dissatisfaction variables that drives the effects. When dropping satisfaction with democracy from the models, the effects are highly similar in direction, with slightly higher levels of significance (see Online Appendix, Table A8).

## Discussion

It is well known that people who are dissatisfied with representative democracy are more supportive of direct democratic practices such as referendums. What is less well known is which kind of dissatisfaction affects support for referendums. By focusing on preferences and perceived evaluations of government responsiveness, we aim to contribute to a better understanding of support for referendums. We build on the literature on representation to distinguish between two core functions of government: listening and leading. If the government is expected to listen rather than lead, support for referendums is higher. To reach the ideal of a close representation of the opinions of the represented, responsive governments, but also referendums, can be appropriate. Second, dissatisfaction with the government's ability to listen to public opinion is associated with higher support for referendums, whereas the opposite is the case for dissatisfaction with the government's ability to lead. If you cannot count on the government to be responsive, direct interventions of citizens might be a way to have policies that better match public opinion. Finally, we find that this relationship is especially strong in countries where referendums occur less frequently in political practice.

There are several key lessons to take from this study. First, it is not the case that general frustration with politics uniformly drives support for referendums. Some kinds of dissatisfaction relate strongly and positively to support for referendums while others might even decrease support. Second, support for alternative models of governance can be understood as a pragmatic solution to specific problems that citizens see with the political system. Those citizens who are dissatisfied due to a deficit in responsiveness consider referendums a good solution, whereas citizens that are dissatisfied due to a deficit in leadership do not. Importantly, this study shows that process preferences should not be studied in isolation but always in connection to the actual context of representative democracy. Depending on what problems citizens see with contemporary politics, they might desire different kinds of institutional reforms to address these problems. Also, the extent to which different decision‐making tools actually fulfill their promise after regular use turns out to shape public preferences for referendums. Whereas more experience with referendums on the country level is not associated with less support for referendums as such, they seem less suitable as a solution to the problem of responsiveness. More in‐depth analysis of country cases or panel studies could provide insights into why this is the case. One potential explanation is that when citizens demand more responsiveness they primarily demand responsiveness to their personal policy positions. As Werner (2019) has recently shown, such instrumental considerations can play a substantial role in shaping support for decision making via referendums. Plausibly, more experience with referendums also shows citizens that they sometimes win and sometimes lose. Yet, it is also possible that citizens are critical of the way referendums are practiced now. They might prefer direct democratic processes that are better integrated in the working of contemporary democracies than is usually the case (Gastil et al. 2014; Chambers 2018).

This study is not without limitations. Most importantly, the observational nature of the data does not allow us to make strong causal claims. Hence, we advocate for future studies that can include experimental or panel data to unpack the causality between dissatisfaction and support for referendums further. Second, we focused on the differential effects of dissatisfaction with leading and listening. However, we want to emphasise that we consider the listening and leading functions of government as only two out of many possible objects of dissatisfaction among citizens that can potentially shape preferences for different democratic reforms. Despite this limitation, our study contributes to the literature on process preferences by taking a first step at disentangling the relationship between dissatisfaction and support for referendums. To further parcel out the dissatisfaction effect, it is advisable for future studies to take representative democracy as a composite system with different institutions and procedures that citizens can be dissatisfied with, rather than relying on broader measures of satisfaction with the functioning of democracy or trust in political institutions.

Dissatisfaction remains a central driver behind support for referendums. However, besides more abstract and value‐oriented considerations about the ability of referendums to counter diffuse disappointment with existing institutions, there are also pragmatic considerations shaping citizens’ views on referendums. In particular, citizens’ practical experience with referendums and the expectation that referendums might address specific problems of political systems also affect popular demand for them. The idea that different citizens might demand different institutional reforms depending on what they believe is wrong with representative democracy should receive greater attention when thinking about potential reforms to representative democracy.

## Supporting information


**Table A1: Democratic norms across representative ideals**
**Table A2: Correlation between representative ideals and democratic norms**
**Table A3: Multilevel regression explaining support for referendums with representation ideals**
**Table A4: Multilevel regression explaining support for referendums by interaction between experience with referendums and dissatisfaction with the listening function ( across different operationalizations of experience with referendums)**
**Table A5: Multilevel regression explaining support for referendums with experience with referendums (across different operationalizations)**
**Table A6: Multilevel regression explaining support for referendums with interactions between indicators for satisfaction and rep. ideals**
**Table A7: Multilevel regression explaining support for referendums with different types of dissatisfaction**
**Table A8: Multilevel regression explaining support for referendums without satisfaction with democracy**

## Notes

1 These respondents are excluded from the main analysis to maintain the same sample sizes across the hypotheses. However, we also run robustness checks that include these cases in the main model, resulting in substantially the same results. Results available from the authors upon request.

2 For more information on correlations between the leader and listener model and democratic norms, see the Online Appendix, Tables A1 and A2.

3 We also run our main analyses without satisfaction with democracy as a control variable. These models yield substantially the same results. See Table A8 in the Online Appendix.
